# Supplementary material for: Deploying an In Vitro Gut Model to Assay the Impact of the Mannan-Oligosaccharide Prebiotic Bio-Mos on the Atlantic Salmon (Salmo salar) Gut Microbiome
Source: Microbiol Spectr. 2022 May 9;10(3):e01953-21. doi: 10.1128/spectrum.01953-21 (PMC9241627; doi:10.1128/spectrum.01953-21)
Supplement: SUPPLEMENTAL FILE 1 — Supplemental material. Download spectrum.01953-21-s001.pdf, PDF file, 1.2 MB [file spectrum.01953-21-s001.pdf]

__Supplementary Table 1__ **Beta diversity and differential abundance values from the comparison of microbial composition between different phases (Pre-Bio-Mos, Bio-Mos and Wash out)** The table summarises different beta-diversity analysis outputs calculated by using different distances: phylogenetic (unweighted, balanced and weighted UniFrac) and ecological (Bray-Curtis and Jaccard's), between different experimental phases: Pre-Bio-Mos, Bio-Mos and Wash out. Numbers represent p-values, with p-values <0.05 identifying statistically significant differences between compared groups. The comparisons are shown for 3 different datasets: (i) All (completed data set containing all the samples sequenced), (ii) a Subset (containing all samples for Pre-Bio-Mos and (iii) the Wash out period, but only stable samplings from Bio-Mos period (time points 22, 24 and 26)). The last row indicates the number of differentially abundant OTUs between Phases of interest.

__Supplementary Figure 1__ **Comparison of key network analysis indicators between different experimental phases (Pre-Bio-Mos, Bio-Mos and Wash out)** Figure compares key characteristics of networks produced for three experimental phases: Pre-Bio-Mos (green), Bio-Mos (red), and Wash out (blue). **A** compares degree of each network; **B** betweenness centrality. The asterisk show significance: (*: $0.01 \leq p < 0.05$; **: $0.05 \leq p < 0.001$; ***: $p \leq 0.001$)

Test		Data	Pre-Bio-Mos vs Bio-Mos	Bio-Mos vs Wash out	Pre-Bio-Mos vs Wash out
UniFrac	Unweighted (0%)	All	0.042	0.029	0.001
		Subset	0.002	0.184	0.001
	Balanced (50%)	All	0.023	0.207	0.001
		Subset	0.007	0.648	0.001
	Weighted (100%)	All	0.022	0.37	0.002
		Subset	0.002	0.717	0.001
Bray-Curtis		All	0.034	0.04	0.001
		Subset	0.003	0.727	0.001
Jaccards		All	0.018	0.05	0.001
		Subset	0.001	0.8	0.001
Differential abundance		Subset	149	5	138

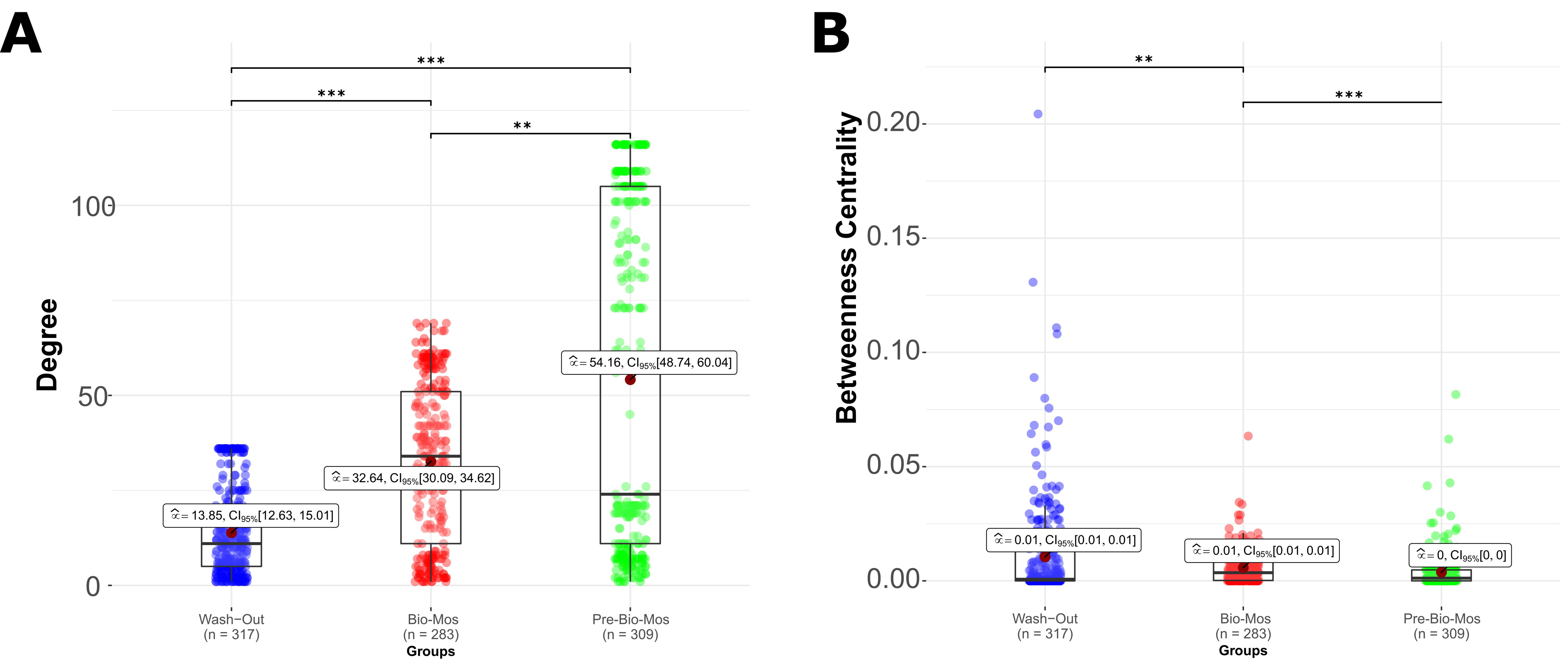
A
B
Degree
Betweenness Centrality
Groups
Wash−Out
(n = 317)
Bio-Mos
(n = 283)
Pre-Bio-Mos
(n = 309)
0
50
100
0.00
0.05
0.10
0.15
0.20
***
**
$\hat{\mu}$ = 13.85, CI95%[12.63, 15.01]
$\hat{\mu}$ = 32.64, CI95%[30.09, 34.62]
$\hat{\mu}$ = 54.16, CI95%[48.74, 60.04]
$\hat{\mu}$ = 0.01, CI95%[0.01, 0.01]
$\hat{\mu}$ = 0, CI95%[0, 0]
